# Increased Transferrin Sialylation Predicts Phenoconversion in Isolated REM Sleep Behavior Disorder

**DOI:** 10.1002/mds.28942

**Published:** 2022-02-07

**Authors:** Ranjani Ganapathy S., Kateřina Levová, Lenka Kotačková, Jiří Trnka, David Zogala, Jan Rusz, Tomáš Zima, David Devos, Karel Šonka, Evžen Růžička, Marta Kalousová, Petr Dušek

**Affiliations:** ^1^ Department of Neurology and Centre of Clinical Neuroscience, First Faculty of Medicine Charles University and General University Hospital in Prague Prague Czech Republic; ^2^ Institute of Medical Biochemistry and Laboratory Diagnostics, First Faculty of Medicine Charles University and General University Hospital in Prague Prague Czech Republic; ^3^ Institute of Nuclear Medicine, First Faculty of Medicine Charles University and General University Hospital in Prague Prague Czech Republic; ^4^ Department of Circuit Theory, Faculty of Electrical Engineering Czech Technical University in Prague Prague Czech Republic; ^5^ Department of Medical Pharmacology, Expert Center for Parkinson, CHU‐Lille, Lille Neuroscience and Cognition, Inserm, UMR‐S1172, LICEND, NS‐Park Network University of Lille Lille France

**Keywords:** Parkinson's disease, rapid eye movement sleep behavior disorder, transferrin, sialylation, phenoconversion

## Abstract

**Background:**

Sialic acid–protein interactions are involved in regulating central nervous system immunity; therefore, derangements in sialylation could be involved in neurodegeneration.

**Objectives:**

We evaluate the differences in serum transferrin sialylation in prodromal and early‐stage Parkinson's disease (PD), its relation to substantia nigra degeneration, and the risk of phenoconversion to manifest disease.

**Methods:**

Sixty treatment‐naive PD patients; 72 polysomnography‐confirmed isolated rapid eye movement sleep behavior disorder (iRBD) patients, that is, patients with prodromal synucleinopathy; and 46 healthy volunteers aged ≥45 years and drinking ≤60 standard drinks per month were included. The proportion of serum low‐sialylated, carbohydrate‐deficient transferrin (CDT) isoforms was assessed using high‐performance liquid chromatography, and the values were adjusted for alcohol intake (CDT_adj_). Dopamine transporter single‐photon emission computed tomography (DaT‐SPECT) imaging was performed. In iRBD, phenoconversion risk of DaT‐SPECT and CDT_adj_ was evaluated using Cox regression adjusted for age and sex.

**Results:**

Median CDT_adj_ was lower in PD (1.1 [interquartile range: 1.0–1.3]%) compared to controls (1.2 [1.1–1.6]%) (*P* = 0.001). In iRBD, median CDT_adj_ was lower in subjects with abnormal (1.1 [0.9–1.3]%) than normal (1.3 [1.2–1.6]%) DaT‐SPECT (*P* = 0.005). After a median 44‐month follow‐up, 20% of iRBD patients progressed to a manifest disease. Although iRBD converters and nonconverters did not significantly differ in CDT_adj_ levels (*P* = 0.189), low CDT_adj_ increased the risk of phenoconversion with hazard ratio 3.2 (*P* = 0.045) but did not refine the phenoconversion risk associated with abnormal DaT‐SPECT yielding hazard ratio 15.8 (*P* < 0.001).

**Conclusions:**

Decreased serum CDT_adj_ is associated with substantia nigra degeneration in synucleinopathies. iRBD patients with low CDT_adj_ are more likely to phenoconvert to manifest disease. © 2022 The Authors. *Movement Disorders* published by Wiley Periodicals LLC on behalf of International Parkinson Movement Disorder Society.

In recent years, the diagnostic approach for Parkinson's disease (PD) has evolved past the classic motor disorder standpoint to a systemic disease with a long latency period when the subclinical synucleinopathy manifests with nonmotor symptoms that can antedate the onset of cardinal motor symptoms by decades.^1^, ^2^, ^3^, ^4^

Isolated rapid eye movement sleep behavior disorder RBD (iRBD), a parasomnia characterized by the loss of the physiological REM sleep skeletal muscle atonia and dream enactment behavior,^5^ is the most specific prodromal symptom of synucleinopathy,^6^ with almost half of those patients converting to PD.^7^, ^8^ In a recent multicenter study, nigroputaminal dopaminergic loss was shown to be the strongest predictor of early phenoconversion.^9^ Yet dopamine transporter single‐photon emission computed tomography (DaT‐SPECT) is an expensive examination associated with radiation exposure, which prevents its usage in close longitudinal monitoring. Finding more convenient blood‐borne markers of early conversion would allow more flexible stratification of patients with imminent conversion.

One of the factors implicated in PD pathophysiology is iron accumulation in substantia nigra, which supposedly accelerates the generation of free radicals and could contribute to a functional impairment of the nigrostriatal system.^10^, ^11^, ^12^ Interestingly, a metanalysis indicated the possibility of iron levels being increased in the cerebrospinal fluid (CSF) and decreased in serum in PD patients compared with controls.^13^ Transferrin is the primary iron‐transporting protein that can also traffic iron away from tissues with its high content.^14^ Many studies on different populations have indicated that transferrin and its receptor gene polymorphisms could modify the risk of PD.^15^, ^16^, ^17^ Thus, understanding transferrin microheterogeneity could provide better insight into PD pathogenesis.

Transferrin heterogeneity is caused by (1) genetic variants; (2) number of iron ions attached; and (3) differences in the carbohydrate moieties dictated by different degrees of branching, terminal sugar residues, and the level of sialylation. Based on differences in sialylation, nine transferrin isotypes containing zero to nine sialic acid residues are recognized.^18^ Transferrins with zero, one, or two sialic acid chains are collectively referred to as carbohydrate‐deficient transferrin (CDT). CDT was found to be elevated in the cerebrospinal fluid and serum of alcoholic patients^19^, ^20^, ^21^, ^22^ and has become an important marker for alcohol use disorder.^23^

Derangements of protein sialylation have been implicated in neurodegenerative disorders. In PD, there is a reduced proportion of monosialylated peripheral IgG.^24^ Differentially sialylated forms of transferrin were also described in PD. When CSF levels were compared, dopaminergic‐treated PD patients had a significantly lower degree of sialylation with a predominance of asialylated form and reduction of tetrasialylated form compared to untreated PD patients and controls. In the serum, the proportion of transferrin isoforms is shifted toward higher sialylated forms in PD patients when compared with controls.^25^ However, it is not known whether altered transferrin sialylation is an early marker of prodromal synucleinopathy or a late marker of fully developed disorder. This study aims to determine whether CDT could be a biomarker for prodromal synucleinopathy, examine its relations with other clinical and imaging parameters, and check whether it can potentially be a predictor of phenoconversion in iRBD.

## Patients and Methods

### Research Participants

Three groups of participants were included: PD patients, iRBD patients, and healthy controls. The PD group consisted of de novo treatment‐naive patients included in the BIO‐PD study; the detailed protocol of this project is described elsewhere.^26^ The diagnosis was confirmed by a movement disorders specialist (P.D.) according to the Movement Disorder Society (MDS) clinical diagnostic criteria.^27^ Exclusion criteria were treatment with antiparkinsonian medication before baseline examination; clinical, imaging, or laboratory signs of atypical parkinsonism; and normal finding on DaT‐SPECT. In the iRBD group, the diagnosis was confirmed by video‐polysomnography according to the International Classification of Sleep Disorders, third edition.^28^ For inclusion, all patients had to be without overt parkinsonism and dementia as well as factors indicating secondary iRBD such as narcolepsy, drug‐induced iRBD (ie, iRBD originating shortly after the initiation of antidepressants), or focal brainstem lesions on magnetic resonance imaging. Control subjects were recruited from the general community through advertisements. To be eligible for the study, controls had to be free of neurologic disorders on interview and examination, active oncologic illness, and abuse of psychoactive substances. All control subjects were examined by a neurologist; iRBD was excluded by thorough history and video‐polysomnography.

Exclusion criteria for all participants were age <45 years, active hepatic illness defined as serum γ‐glutamyltransferase levels above 3 μkat/L, and alcohol abuse defined as drinking more than 60 standard drinks (ie, 500 mL of beer, 200 mL of wine, or 50 mL of liquor) per month. This cutoff for alcohol intake was chosen based on the published observations that CDT increases in people drinking more than 50 to 80 g of alcohol (ie, approximately two standard drinks) per day.^29^ Clinical examinations and blood sampling were performed at baseline visit, and DaT‐SPECT was performed within 1 month after the baseline visit. This study was approved by the institutional ethics committee of the General University Hospital in Prague (approval numbers: 110/14 and 11/15 for PD and RBD patients, respectively); all participants were informed about the study and provided written informed consent.

### Clinical Assessment

The clinical evaluation of each participant included a structured interview gathering information on personal and medical history, history of drug and substance intake, and current drug usage. All participants were examined using Movement Disorder Society‐Unified Parkinson's Disease Rating Scale (MDS‐UPDRS)^30^ by an MDS‐certified rater. For cognitive evaluation, Montreal Cognitive Assessment (MoCA)^31^ was used. Olfaction was investigated using the University of Pennsylvania Smell Identification Test (UPSIT).^32^ Autonomic functions were assessed using the Scales for Outcomes in PD‐Autonomic (SCOPA‐AUT) questionnaire.^33^, ^34^

After the baseline examination, iRBD patients were followed every 12 months by a neurologist with expertise in movement disorders (P.D.). Follow‐up visits were performed according to predefined protocol, including comprehensive neurological examination, detailed clinical history, and MDS‐UPDRS and MoCA scoring. Phenoconversion was recorded upon the occurrence of parkinsonism (defined as bradykinesia and at least one of rigidity or resting tremor)^27^ and/or dementia (defined as functional impairment in activities of daily living reported by the patient and/or caregiver and evidence of cognitive impairment on MoCA testing).^35^

### DaT‐SPECT

DaT‐SPECT was performed in PD and RBD patients, but not in controls, using the [123I]‐2‐b‐carbomethoxy‐3b‐(4‐iodophenyl)‐*N*‐(3‐fluoropropyl) nortropane ([123I]FP‐CIT, DaTSCAN, GE Healthcare, Chicago, IL) tracer according to the European Association of Nuclear Medicine procedure guidelines^36^; the detailed protocol is described elsewhere.^37^ Automated semiquantitative analysis was performed using the BasGan V2 software, and specific binding ratios (SBR) in both putamina relative to background binding were calculated; the lower value from both hemispheres was used for further analyses. SBR values below the 95% single‐sided prediction interval for linear regression of age and putamen SBR in a group of controls obtained from a reference database were considered abnormal.^37^, ^38^ Furthermore, sensitivity analysis with a lower cutoff for abnormal DaT‐SPECT, that is, below the 90% prediction interval, was performed.

### Laboratory Analysis

Venous blood samples from all participants were drawn in the morning after an overnight fast into test tubes without anticoagulant. Sera were separated within 60 minutes after blood collection using centrifugation of blood at 1450 g for 10 minutes and then immediately aliquoted and frozen at −80°C until further biochemical analysis.

CDT was measured using high‐performance liquid chromatography method on a Variant analyzer (Bio‐Rad Laboratories GmbH, Munich, Germany) using standard kits (Ready‐Prep %CDT by HPLC TM, Bio‐Rad Laboratories GmbH). Sample preparation and measurement was performed according to the manufacturer's protocol. Chromatogram was evaluated by software Variant (Bio‐Rad Laboratories GmbH). %CDT was calculated using the following equation: %CDT = disialotransferrin + asialotransferrin area/total transferrin area × 100. The percentages of other isoforms of transferrin (trisialotransferrin, tetrasialotransferrin, and pentasialotransferrin) were calculated from the total transferrin area as well.

Basic biochemical analyses were performed in the laboratory of the Institute of Medical Biochemistry and Laboratory Diagnostics on automated analyzers. Serum ferritin was measured by chemiluminescence immunoassay on an Atellica analyzer (Siemens Healthineers, Erlangen, Germany). Serum transferrin was analyzed by immunoturbidimetric assay using Cobas 8000 system (Roche Diagnostic, Mannheim, Germany). The latter analyzer was also used for serum glucose determination by the hexokinase method.

### Statistics

Distribution of variables was checked by visual analysis of histograms and Q–Q plots and by formal testing using the Kolmogorov–Smirnov test. Between‐group comparisons were performed either using the univariate general linear model with the group as a fixed factor and age and sex as covariates or using the Kruskal–Wallis test as appropriate. Post hoc tests were performed using Fisher's least significant difference or Dunn's test. χ^2^ test was used to analyze binary variables. Depending on data distribution, Pearson's or Spearman's correlation coefficients were calculated to examine the relationships between biochemical and clinical variables.

Survival Kaplan–Meier analyses were conducted to estimate the conversion risk for CDT, DaT‐SPECT, and motor and olfactory scores. For survival analyses, continuous variables were dichotomized. Mild motor impairment was defined as MDS‐UPDRS III score >6 excluding postural and action tremor items.^4^ Hyposmia was defined as UPSIT score ≤25 (ie, severe microsmia or anosmia according to the UPSIT scoring manual).^32^ The cutoff for CDT values was identified by the Youden method; that is, receiver operating characteristic (ROC) analysis was performed, and the cutoff value with the highest sum of sensitivity and specificity for the discrimination of converters and nonconverters was determined. Censoring time was set at the time of last assessment for nonconverters and at the time of conversion for converters. Hazard ratios for each variable were calculated, with Cox regression adjusted for age and sex. IBM SPSS statistics version 25 (IBM, Armonk, NY) was used for statistical analysis; graphs were plotted using Graphpad Prism, version 9.0 (Graphpad Software, San Diego, CA).

## Results

**FIG 1:**
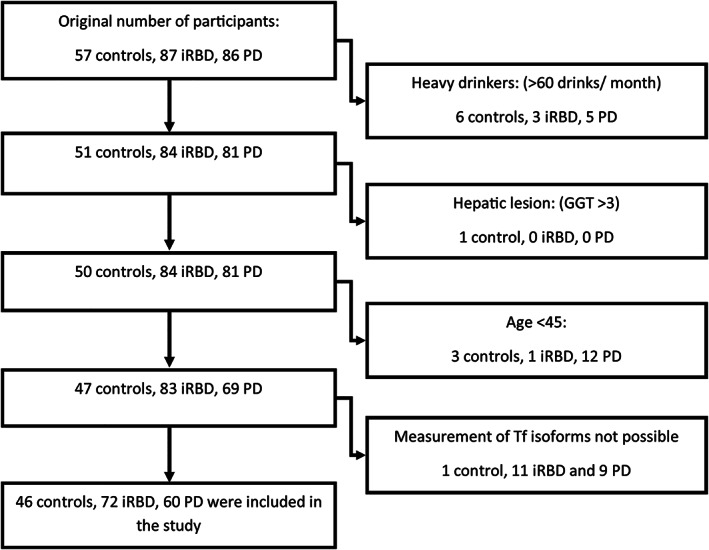
Flow diagram of patient inclusion. In PD patients, serum was not available for 8, and genetic Tf variant precluded interpretation of CDT (carbohydrate‐deficient transferrin) analysis in 1 participant. In RBD (rapid eye movement sleep behavior disorder) patients, serum was not available for 10, and genetic Tf variant precluded interpretation of CDT analysis in 1 participant. In 1 control subject, Tf analysis was uninterpretable due to signal interference.

From all eligible subjects, 60 treatment‐naive PD patients, 72 iRBD patients, and 46 healthy controls were included in the study per the flow diagram shown in Figure 1.

### Comparison of Patients and Controls

The comparison of demographic, clinical, and biochemical parameters in patients and controls is presented in Table 1. Groups did not differ significantly in age or alcohol intake, although the iRBD group was slightly older (*P* = 0.13) and consumed less alcohol (*P* = 0.061 for men and 0.053 for women) than controls. A difference in the sex distribution was observed, with the men/women ratio being higher for the iRBD group compared to PD and controls (*P* = 0.001). There were no between‐group differences in the prevalence of diabetes, body mass index, serum transferrin, ferritin, and glucose levels. There were significant differences in clinical variables related to symptoms of synucleinopathy—MDS‐UPDRS, Part III (*P* < 0.001), SCOPA‐AUT (*P* < 0.001) and UPSIT scores (*P* < 0.001), and DaT‐SPECT (*P* < 0.001).

**TABLE 1:** Demographic, clinical, and biochemical parameters in PD and RBD patients and controls

	PD (n = 60)	RBD (n = 72)	Controls (n = 46)	Uncorrected *P*‐value	Post hoc tests
Demography					
Males/females (n)	37/23	64/8	34/12	**0.001** ^a^	n.d.
Age (y)^b^	65.4 ± 9.3	67.1 ± 7.3	63.9 ± 8.7	0.133^c^	n.d.
Symptom duration (y)^b^	1.7 ± 1.8	6.1 ± 5.6	n.d.	n.d.	n.d.
Standard alcoholic drinks per month (n)^d^	Males 20 (10–30) Females 3 (0–7)	Males 11 (5–22) Females 1 (0–6)	Males 21 (9–30) Females 8 (2–19)	Males 0.061^e^ Females 0.053^e^	n.d.
BMI^b^	28.0 ± 4.1	28.3 ± 4.3	27.6 ± 4.1	0.639^c^	n.d.
Diabetic/nondiabetic	4/56	12/60	5/41	0.202^a^	n.d.
Clinical parameters					
MDS‐UPDRS III^d^	29 (21–40)	6 (2–9)	3 (1–5)	**<0.001** ^e^	PD >>> RBD
					PD >>> controls
MoCA^b^	24.7 ± 3.1	23.8 ± 2.9	25.1 ± 2.4	0.249^c^	n.d.
UPSIT^b^	20.8 ± 6.1	23.2 ± 7.6	31.8 ± 4.5	**<0.001** ^c^	Controls >>> RBD
					Controls >>> PD RBD > PD
SCOPA‐AUT^b^	9.8 ± 6.7	12.5 ± 8.0	6.0 ± 3.9	**<0.001** ^c^	RBD >>> controls RBD > PD
Putaminal SBR^b^	1.5 ± 0.4	2.8 ± 0.7	n.d.	**<0.001** ^c^	n.d.
Biochemical parameters					
Serum ferritin (μg/L)^d^	Males 166.9 (90.9–225.9) Females 107.4 (63.6–154.7)	Males 169.8 (95.8–279.9) Females 69.1 (50.0–157.4)	Males 124.0 (89.2–209.8) Females 101.3 (30.9–139.6)	Males 0.351^e^ Females 0.590^e^	n.d.
Serum transferrin (g/L)^b^	2.5 ± 0.3	2.5 ± 0.3	2.6 ± 0.3	0.422^c^	n.d.
Serum glucose (mmol/L)^d^	5.3 (5.0–5.7)	5.2 (4.8–5.9)	5.3 (4.9–5.8)	0.403^e^	n.d.
CDT_adj_ (%)^d^	1.1 (1.0–1.3)	1.3 (1.1–1.5)	1.2 (1.1–1.6)	**0.001** ^e^	Controls >> PD
					RBD >> PD

Significant differences are marked with bold text. Post hoc test results are as follows: “>>>” = *P* < 0.001, “>>” = *P* < 0.01, “>” = *P* < 0.05. Please note that due to nonnormal (heavy‐tailed) data distribution and significant effect of sex, the number of standard alcoholic drinks per month and serum ferritin concentration are shown separately for men and women.

^a^ χ^2^ test.

^b^ Values reported as mean ± standard deviation.

^c^ Analysis of covariance with *P*‐values adjusted for age and sex with post hoc least square difference test (one‐way analysis of variance was used for age comparison).

^d^ Values reported as median (interquartile range).

^e^ Kruskal–Wallis test with post hoc Dunn's test.

Abbreviations: PD, Parkinson's disease; RBD, rapid eye movement sleep behavior disorder; n, number; n.d., not done; BMI, body mass index; MDS‐UPDRS, Movement Disorder Society‐Unified Parkinson's Disease Rating Scale; MoCA, Montreal Cognitive Assessment; UPSIT, University of Pennsylvania Smell Identification Test; SCOPA‐AUT, Scales for Outcomes in Parkinson Disease‐Autonomic; CDT_adj_, carbohydrate‐deficient transferrin adjusted for alcohol intake; SBR, specific binding ratio.

Despite excluding subjects who consumed >60 standard drinks per month, the effect of alcohol intake on CDT was significant in all groups (*P* < 0.01). We therefore adjusted CDT values for alcohol intake (Supplementary Fig. S1). CDT adjusted for alcohol intake (CDT_adj_) was lower in PD compared to controls and iRBD patients (*P* = 0.001) (Table 1). Tri‐, tetra‐ (the major transferrin isoform), and pentasialotransferrins did not have significantly different ratios in controls and iRBD and PD patients.

### Relationship between CDT, Nigrostriatal Degeneration, and Clinical Variables in Synucleinopathies

CDT_adj_ correlated with the putaminal SBR (r_s_ = 0.29, *P* = 0.02) in the RBD group, whereas significant correlation was not observed in the PD group (r_s_ = 0.10, *P* = 0.44). In the merged group of synucleinopathy patients (ie, PD and iRBD), moderate correlation between CDT_adj_ and putaminal SBR was observed (r_s_ = 0.35, *P* < 0.001) (Supplementary Fig. S2). CDT_adj_ was not related to other clinical variables, including MDS‐UPDRS III, MoCA, SCOPA‐AUT, and UPSIT scores or serum ferritin, transferrin, or glucose levels in any group.

**FIG 2:**
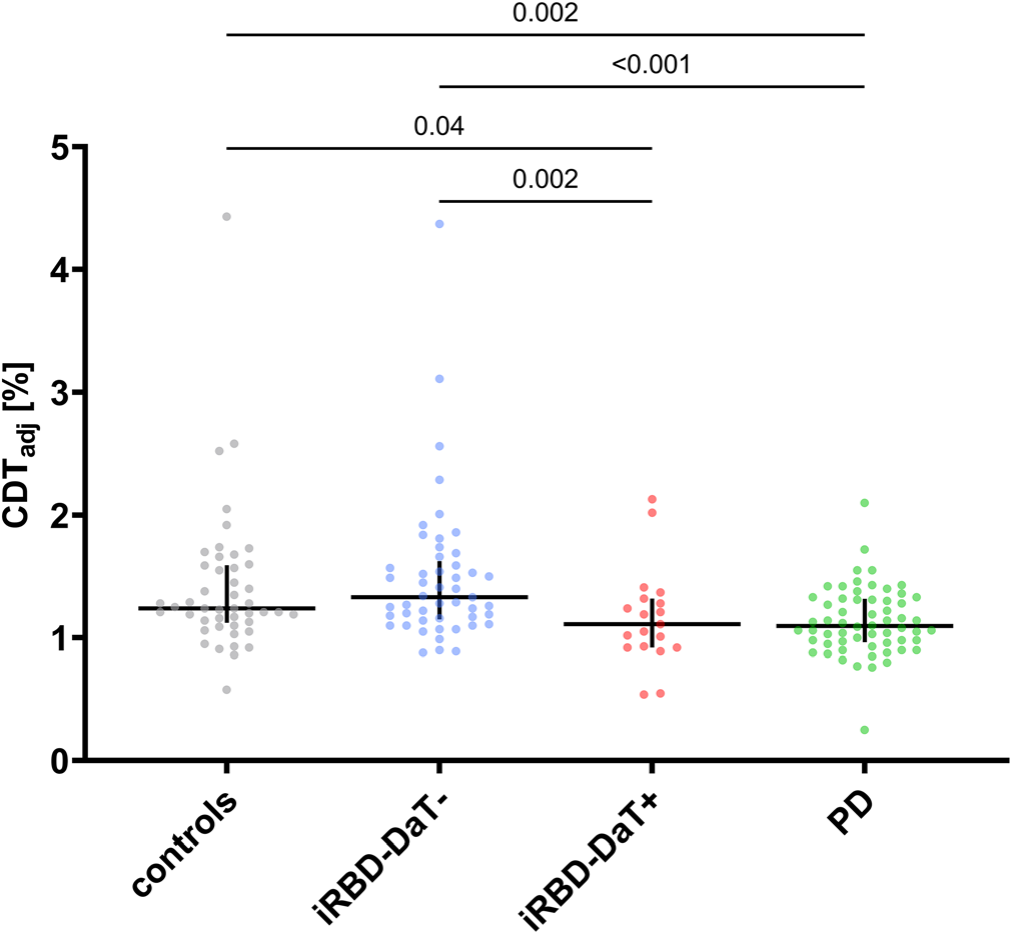
Scatter plots of CDT_adj_ illustrating that CDT_adj_ in iRBD (isolated rapid eye movement sleep behavior disorder) with normal DaT‐SPECT (iRBD‐DaT−) is comparable to that in controls, whereas CDT_adj_ in iRBD with abnormal DaT‐SPECT (iRBD‐DaT+) is comparable to that of PD. Significant *P*‐values of Dunn's post hoc pairwise comparisons are shown. Horizontal/vertical lines represent medians/interquartile ranges. CDT, carbohydrate‐deficient transferrin. DaT‐SPECT, dopamine transporter single‐photon emission computed tomography. [Color figure can be viewed at wileyonlinelibrary.com]

In a post hoc analysis, iRBD patients were divided into subgroups with normal (iRBD‐DaT–) and abnormal (iRBD‐DaT+) DaT‐SPECT. DaT‐SPECT was abnormal in 19 (26.4%) iRBD patients. CDT_adj_ in iRBD‐DaT+ (median 1.1 [interquartile range, IQR, 0.9–1.3]%) was lower compared to iRBD‐DaT– (1.3 [IQR 1.2–1.6]%) (*P* = 0.002) and controls (1.2 [IQR 1.1–1.6]%) (*P* = 0.039), whereas it was comparable to that of PD (1.1 [IQR 1.0–1.3]%) (*P* = 0.868) (Fig. 2).

### CDT as a Predictor of Phenoconversion in iRBD

Six iRBD patients (8%) had no follow‐up visit (1 died and 5 refused to participate in longitudinal observation) and were excluded from the survival analysis. From the remaining 66 iRBD patients, 13 (20%) developed an overt synucleinopathy for an average follow‐up time of 41.9 ± 19.6 (median = 44.0) months. Nine patients developed parkinsonism, 2 dementia, and 2 dementia and parkinsonism. Compared to iRBD nonconverters, iRBD converters were older (*P* = 0.043), had longer symptom duration (*P* = 0.013), higher MDS‐UPDRS III score (*P* = 0.026), lower UPSIT score (0.045), and more severe nigroputaminal deficit on DaT‐SPECT (*P* < 0.001). On the contrary, iRBD converters did not have significantly different CDT_adj_ values compared to nonconverters (*P* = 0.189) (Supplementary Table S1).

**FIG 3:**
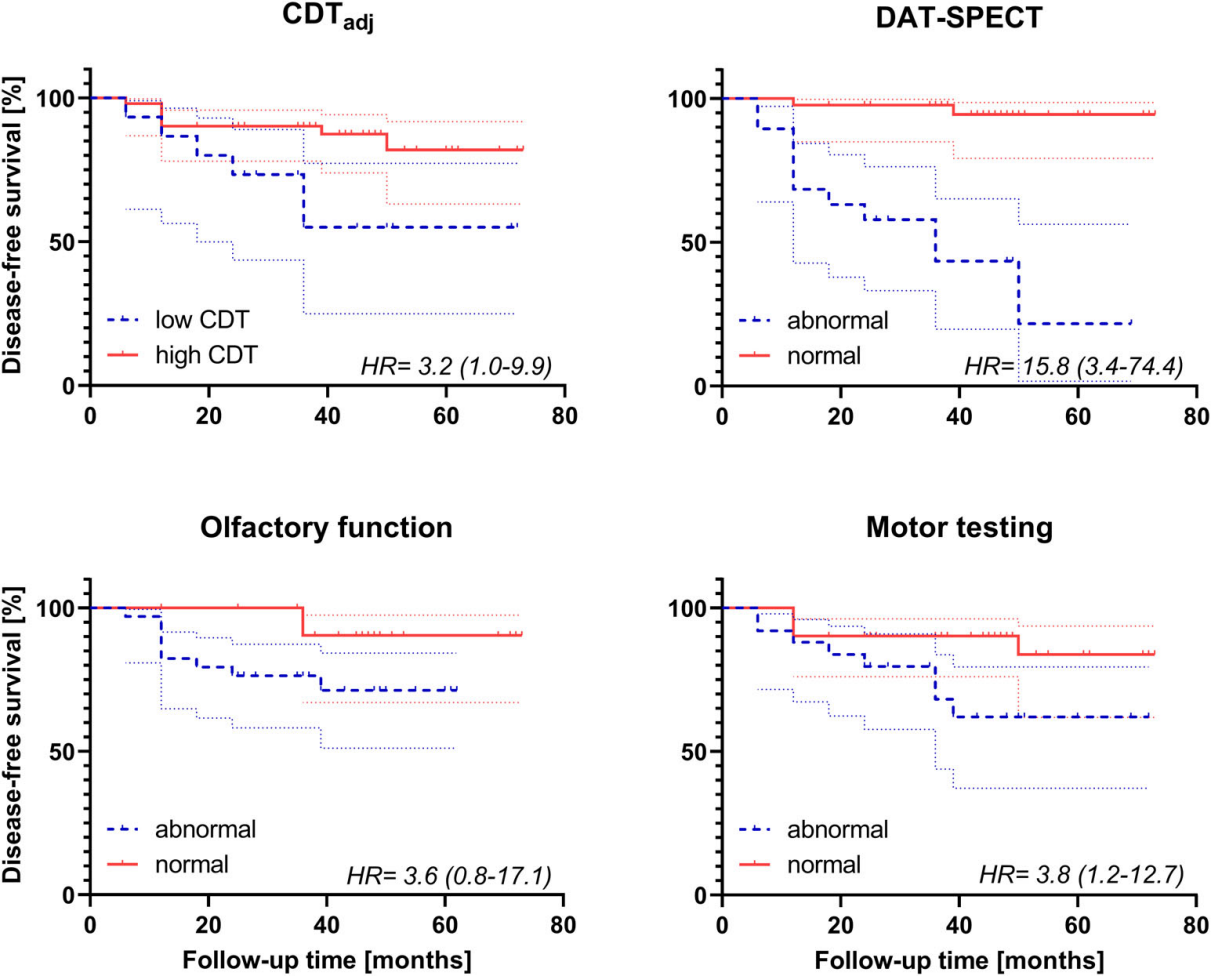
Kaplan–Meier plots of disease‐free survival of patients with iRBD stratified according to alcohol intake‐adjusted CDT (low <1.060%; high ≥1.060%), DaT‐SPECT (abnormal vs. normal) findings, olfactory function (UPSIT score abnormal ≤25; normal >25), and motor testing (MDS‐UPDRS III score excluding postural and action tremor items normal ≤6; abnormal >6). Dotted lines represent 95% confidence bands. Hazard ratios calculated using Cox proportional hazards regression analysis adjusted for age and sex with 95% confidence intervals in parentheses are shown. CDT, carbohydrate‐deficient transferrin; DaT‐SPECT, dopamine transporter single‐photon emission computed tomography; HR, hazard ratio; iRBD, isolated rapid eye movement sleep behavior disorder; MDS‐UPDRS, Movement Disorder Society‐Unified Parkinson's Disease Rating Scale; UPSIT, University of Pennsylvania Smell Identification Test. [Color figure can be viewed at wileyonlinelibrary.com]

Using ROC, the optimal CDT_adj_ cutoff maximizing discrimination between iRBD converters and nonconverters was calculated as 1.060%. Using this cutoff, when CDT_adj_ was considered as a binary variable, iRBD patients with CDT <1.060% have a significantly increased risk of phenoconversion with a hazard ratio of 3.2 (*P* = 0.045; 95% confidence interval [CI] 1.0–9.9) compared to those with CDT ≥1.060%. In comparison, for iRBD patients with abnormal DaT‐SPECT, the hazard ratio of phenoconversion was 15.8 (*P* < 0.001, 95% CI 3.4–74.4) (Fig. 3). Next, models including the combination of DaT‐SPECT and CDT and including DaT‐SPECT only were compared using multivariate backward stepwise Cox regression. The simpler model including only DaT‐SPECT was not inferior and was therefore preferred. CDT thus does not refine the estimation of phenoconversion risk in addition to DaT‐SPECT results (Table 2). In addition, mild motor impairment was significantly associated with increased risk for phenoconversion with HR 3.8 (*P* = 0.027; 95% CI 1.2–12.7). Olfactory impairment did not significantly increase the risk of phenoconversion (HR 3.6; *P* = 0.103; 95% CI 0.8–17.1) (Fig. 3).

**TABLE 2:** Multivariate backward stepwise Cox regression with a combination of DaT‐SPECT and CDT and only DaT‐SPECT

Variable	Coefficient	SE	HR	95% CI	*P*‐value
Step 1					
DaT‐SPECT	2.7	0.8	14.7	3.0–71.6	**<0.001**
CDT	0.3	0.6	1.4	0.4–4.8	0.581
Step 2					
DaT‐SPECT	2.8	0.8	15.8	3.4–74.4	**<0.001**

Significant differences are marked with bold text. Values are adjusted for age and sex.

Abbreviations: DaT‐SPECT, dopamine transporter single‐photon emission computed tomography; CDT, carbohydrate‐deficient transferrin; SE, standard error; HR, hazard ratio; CI, confidence interval.

### Secondary and Sensitivity Analyses

In iRBD, patients excluded for the lack of CDT values had lower MoCA score (*P* = 0.004) and less‐severe nigroputaminal deficit on DaT‐SPECT (*P* = 0.013). In PD, patients excluded for the lack of CDT values had lower UPSIT score (*P* = <0.001). Other variables were not significantly different between included and excluded participants (Supplementary Table S2).

When lower cutoff for abnormal DaT‐SPECT (ie, 90% prediction interval) was applied, DaT‐SPECT was abnormal in 34 (47.2%) iRBD patients. CDT_adj_ in iRBD‐DaT+ (1.2 [IQR 1.0–1.4]%) was lower compared to iRBD‐DaT– (1.4 [IQR 1.2–1.6]%) (*P* = 0.007) but not significantly different compared to controls (*P* = 0.142). Hazard ratio of phenoconversion for patients with abnormal DaT‐SPECT was 5.8 (*P* = 0.021, 95% CI 1.3–25.9) and was thus substantially lower compared to the more stringent DaT‐SPECT cutoff (Supplementary Fig. S3).

Using raw CDT values instead of CDT_adj_ values did not substantially affect between‐group comparisons or the hazard ratio of phenoconversion. For example, using the optimal raw CDT cutoff 1.165% yielded the hazard ratio for phenoconversion of 5.4 (*P* = 0.011, 95% CI 1.5–19.4) in iRBD patients with CDT <1.165% (Supplementary Fig. S4).

## Discussion

In this study, we assessed serum transferrin sialylation isoform profiles in de novo treatment‐naive PD and prodromal synucleinopathy (iRBD) patients and in controls and their relations to clinical and imaging parameters. We found that compared to controls, the proportion of serum CDT is lower in PD and in iRBD patients with abnormal DaT‐SPECT. In addition, iRBD patients with low serum CDT were found to have increased risk for phenoconversion.

Our findings are in concordance with the study by van Kamp and colleagues,^25^ where they similarly reported that the proportion of transferrin isoforms in the serum is shifted toward higher‐sialylated forms in PD patients compared with controls. We have additionally shown that disrupted transferrin sialylation is already apparent in the prodromal disease, at the stage when nigral dopaminergic neurons are affected by the neurodegenerative process. Another study evaluating the profiles of serum samples from iRBD patients found that the parameter corresponding to the amount of *N*‐acetylneuraminic acid (the most abundant form of sialic acid in human cells) in glycoproteins was increased in patients after the diagnosis of dementia with Lewy bodies (DLB) compared to the pre‐DLB stage but was higher in controls than pre‐DLB. These findings are in apparent contradiction to results in our iRBD patients converting predominantly to PD but generally support altered sialylation in synucleinopathies.^39^ Reductions in sialylation of *N*‐ and *O*‐linked glycans were reported in PLA2G6‐associated neurodegeneration, which may manifest as early‐onset PD, although this was found to be due to disruption in Golgi morphology causing a global reduction in *O*‐linked glycans, not due to a decrease in the process of sialylation.^40^ There is also a reduction in sialylation of the peripheral IgG glycome in PD compared to controls, which may contribute to a reduced capacity for IgG to inhibit Fc receptors and cause a state of low‐grade inflammation in PD.^24^ Given all these findings, it is likely that abnormal sialylation is not limited to serum transferrin but more likely reflects deeper derangement of posttranslational protein modifications in PD.

Cell‐surface sialylation acts as an immune checkpoint.^41^ Sialic acid–binding immunoglobulin‐type lectins (Siglecs) expressed on leukocytes recognize sialic acid–containing glycans as ligands. Microglial Siglec receptors inhibit microglial activation, inflammation, phagocytosis, and oxidative burst.^41^ Siglec‐E knock‐out mice demonstrated higher oxidative damage,^42^ and the deletion of Siglec‐E's counterbalancing receptor led to decreased age‐related inflammatory signs and neuronal loss.^43^ Higher systemic inflammatory activity was described in PD^44^, ^45^ and the death of dopaminergic neurons in PD was associated with the activation of inflammatory microglial phenotypes that facilitate the progression of the disease.^46^ Hyperactivity of these microglia could possibly be limited by Siglec‐E interaction with sialic acids. Another parallel could be drawn in that sialylated IgGs are less capable of ligating to Fcγ‐RIIIa receptors on natural killer cells, leading to lower levels of inflammation.^47^ Thus, protein hyposialylation could hypothetically explain how immunological derangements may be related to neurodegeneration. On the contrary, weakening of the sialylation axis may also have beneficial effects.^41^ The loss of function of the lysosomal sialidase NEU1 leads to an Alzheimer's disease (AD)‐like process in mice due to the accumulation of hypersialylated amyloid precursor protein in lysosomes and the extracellular release of amyloid β peptides.^48^ This suggests that sialylation of the amyloid precursor protein prevents its degradation and could lead to the progression of AD. Similar findings were observed in sialylation‐deficient mutant (Lec‐2) cells, which secreted half as much amyloid β peptide as wild‐type cells.^49^ In AD patients, transferrin C1 homozygotes (as opposed to those who carry the more proinflammatory C2 allele) had increased levels of glycosylation. Transferrin C2 allele is associated with earlier‐onset AD,^50^ and its presence is associated with lower amounts of the higher‐sialylated forms of transferrin.^51^, ^52^ Interestingly, hypersialylation also plays a role in tumor progression and metastasis as sialyltransferases support cell migration and invasion and cancer cell survival, evading detection by the immune system, and may also contribute to drug resistance.^53^

Comparative proteomic analysis of serum samples between iRBD patients and controls has indicated differences between the groups in their serum glycomes and *N*‐glycan profiles.^54^ It was also found that in iRBD patients, there was dysregulation of several proteins playing a role in immunity, inflammation, cholesterol transport, complement activation, and blood coagulation.^55^

Based on our results, the hazard ratio of phenoconversion for CDT was comparable to previously identified risk factors, hyposmia and mild motor impairment.^8^ CDT may thus become inexpensive and a routinely available serum biomarker of phenoconversion in iRBD. Yet several factors may bias CDT measurement, including pregnancy, usage of oral contraceptive pills, liver disease, severe iron deficiency, and alcohol abuse.^56^ Alcohol abuse is associated with poorer activity of serum glycosyltransferases leading to reduced carbohydrate moieties on glyconjugates, therefore causing an elevation in CDT.^20^, ^57^ Importantly, our calculations with values adjusted and unadjusted for alcohol intake suggest that the association between serum CDT and nigrostriatal degeneration is only minimally affected by alcohol in persons not drinking excessively. Interestingly, despite the fact that alcohol affects protein sialylation in the opposite direction as PD, there seems to be no association between alcohol intake and PD risk.^58^

The predictive power of CDT for phenoconversion appears to be related to its association with dopaminergic nigroputaminal degeneration. As serum CDT does not provide added value to DaT‐SPECT, it can be useful when the latter examination is not available. Consistent with our findings, abnormal DaT‐SPECT was shown as the strongest predictor for phenoconversion, but our hazard ratio is much higher than that previously reported.^8^, ^9^ This might be due to several reasons. First, we have a relatively stringent threshold for abnormal DaT‐SPECT, that is, below 95% age‐specific prediction interval, which is equivalent to the cutoff of 2 SD (standard deviation) below population mean,^59^ whereas it was set as 1.5 SD below the mean of control subjects in other recent studies.^9^, ^60^ Therefore, iRBD subjects considered to have abnormal DaT‐SPECT in our study may have more severe nigrostriatal degeneration compared to the latter studies. When the cutoff was lowered to 90% prediction interval, the hazard ratio for phenoconversion decreased to 5.8, which is comparable to previously published values. Second, most of our patients converted to parkinsonism, and abnormal DaT‐SPECT is a better marker of parkinsonism than dementia. Third, our follow‐up duration was shorter than that of previous studies, and therefore, we found conversion only in those iRBD patients who already had DaT‐SPECT signs of nigrostriatal degeneration at baseline and were on the verge of phenoconversion. With longer follow‐up, it can be expected that patients with initially normal DaT‐SPECT would develop nigrostriatal degeneration and manifest disease.

The main limitation of the present study is the relatively short follow‐up duration in assessing phenoconversion, and therefore, the hazard ratios are not generalizable. In addition, 13% of iRBD patients could not be included in the analysis due to lack of CDT measurements, and sensitivity analysis showed more cognitive impairment and less‐severe nigroputaminal dopaminergic degeneration in excluded patients, admitting minor selection bias. We also had a lower sample size of iRBD patients compared to previous multicentric studies, and measurements in CSF were not performed. Measuring CDT longitudinally in serum and CSF would have provided more clarity on the evolution of transferrin isoforms over time and would have helped better understand the mechanisms it plays within the brain.^61^ CDT as a marker may become more relevant with longitudinal intraindividual follow‐up.

In conclusion, decreased serum CDT is associated with substantia nigra degeneration in prodromal and manifest synucleinopathies and may be a surrogate marker of nigral dopaminergic loss and therefore of phenoconversion. In a broader view, our results indicate that deranged protein sialylation may play a role in the pathophysiology of synucleinopathies and should be further investigated. Future studies in prodromal synucleinopathies with longer follow‐up should include serial measurement of sialylated isoforms of transferrin and other proteins to better understand the time frame, extent, and mechanisms of abnormal protein sialylation in PD and other synucleinopathies.

## Full financial disclosures for the previous 12 months

David Zogala received consultancy honoraria from Pfizer, Bracco, and Novartis. Petr Dušek received funding from the General University Hospital (grant no. GIP‐20‐L‐13‐212) and honoraria for clinical trials from Alexion Pharmaceuticals. Karel Šonka received grants from the Czech Ministry of Health, advisory board payment from UCB Pharma, honoraria for clinical trials from UCB Pharma and Avadel Pharmaceuticals, and speaker fee from Sanofi. Ranjani Ganapathy S., Kateřina Levová, Lenka Kotačková, Jiří Trnka, Jan Rusz, Tomáš Zima, David Devos, Evžen Růžička, and Marta Kalousová have nothing to disclose.

## Author Roles

(1) Research project: A. Conception, B. Organization, C. Execution; (2) Statistical analysis: A. Design, B. Execution, C. Review and critique; (3) Manuscript preparation: A. Writing of the first draft, B. Review and critique.

R.G.: 1C, 2A, 2B, 3A

K.L.: 1C, 2C, 3B

L.K.: 1A, 1B, 2C, 3B

J.T.: 1B, 1C, 2C, 3B

D.Z.: 1B, 1C, 2C, 3B

J.R.: 1A, 2B, 2C, 3B

T.Z.: 1C, 2C, 3B

D.D.: 1A, 2C, 3B

K.Š.: 1A, 1B, 1C, 2C, 3B

E.R.: 1A, 1B, 1C, 2C, 3B

M.K.: 1C, 2C, 3B

P.D.: 1A, 1B, 1C, 2A, 2B, 3A

## Supporting information


**APPENDIX S1.** Supporting Information

## Data Availability

The data that support the findings of this study are available from the corresponding author upon reasonable request.
