# Supplementary material for: Increased Transferrin Sialylation Predicts Phenoconversion in Isolated REM Sleep Behavior Disorder
Source: Mov Disord. 2022 Feb 7;37(5):983–92. doi: 10.1002/mds.28942 (PMC9305135; doi:10.1002/mds.28942)
Supplement: Supplementary file 1 — APPENDIX S1. Supporting Information [file MDS-37-983-s001.docx]

**Supplementary Fig.1** Dependence of serum CDT on alcohol intake during the last month

**
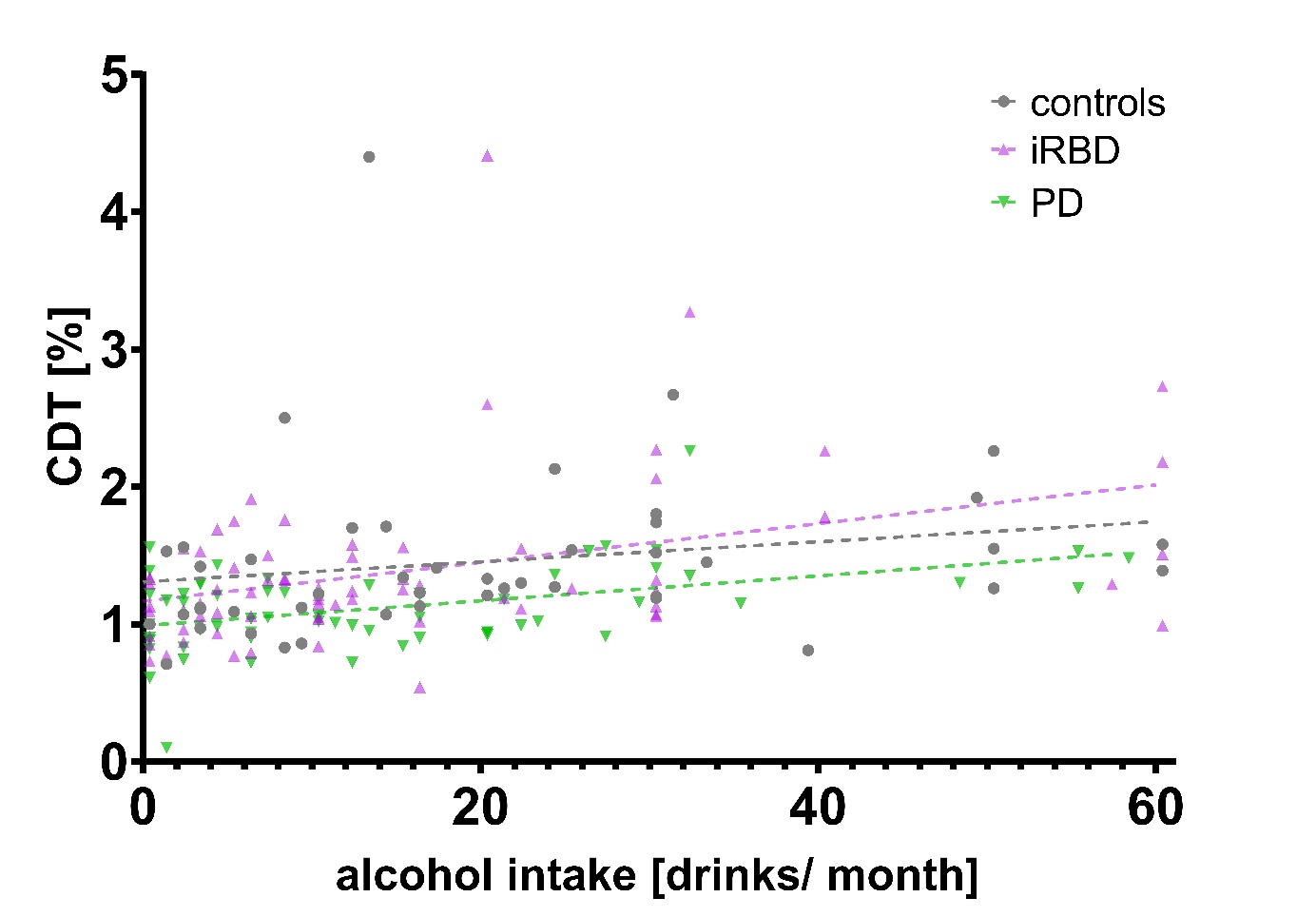
**

|  | **Spearman correlation coefficient (r_s_)** | **p-value** | **Linear regression equation** |
| --- | --- | --- | --- |
| **Controls** | 0.42 | 0.004 | Y = 0,0073*X + 1,307 |
| **iRBD** | 0.36 | 0.002 | Y = 0,0141*X + 1,167 |
| **PD** | 0.38 | 0.003 | Y = 0,0091*X + 0,989 |

Graph and Table show significant positive association between alcohol intake and CDT (%) values in PD, iRBD, and control groups. Dashed linear regression fit lines for each group are shown; corresponding regression equations are shown in the table. We did not find differences in regression slopes for individual groups (F_2,172_ = 0.79, p = 0.45). The pooled slope for all three groups equaling 0.011 CDT (%) increase for each consumed standard drink was used to adjust CDT values for alcohol intake. There was a significant effect of group on the Y-intercept (F_2,174_ = 5.55, p = 0.005) which was lower in the PD compared to control and RBD group.

**Supplementary Fig.2** Association between DAT-SPECT and CDT_adj_ in iRBD and PD groups

**
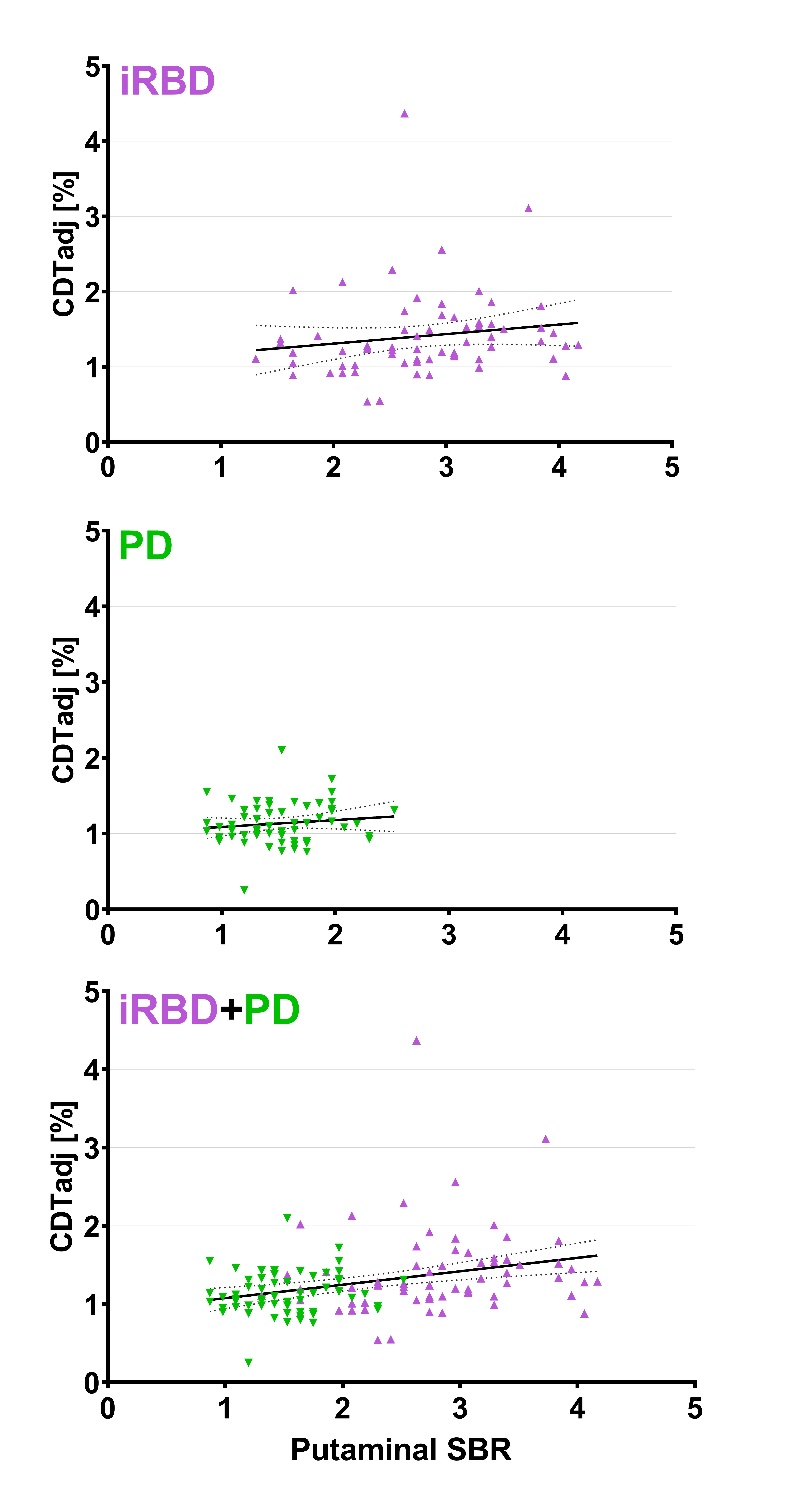
**

|  | **Spearman correlation coefficient (r_s_)** | **p-value** | **Linear regression equation** |
| --- | --- | --- | --- |
| **iRBD** | 0.29 | 0.02 | Y = 0,1269*X + 1,055 |
| **PD** | 0.10 | 0.44 | Y = 0,09093*X + 0,9977 |
| **iRBD+PD** | 0.35 | <0.001 | Y = 0,1717*X + 0,9041 |

Graph and Table show association between lower putaminal SBR from both hemispheres and CDT_adj_ (%) values. Separate graphs are shown for iRBD (upper graph, violet triangles), PD (middle graph, green triangles) and merged iRBD+PD (bottom graph) groups. Linear regression fit lines with 95% confidence bands are shown; corresponding regression equations are shown in the table. We did not find differences in regression slopes (F_1,122_ = 0.04, p = 0.84) and Y-intercepts (F_2,123_ = 0.99, p = 0.32) for PD and iRBD groups indicating that single linear regression model provides best fit for merged iRBD+PD data.

**Supplementary Table 1:** Demographic, clinical, and biochemical parameters in iRBD converters and iRBD non-converters

|  | **iRBD converters (n=13)** | **iRBD non-converters (n=53)** | **Uncorrected  p-value** |
| --- | --- | --- | --- |
| **Demography** | | | |
| Males/females [n] | 11/2 | 47/6 | 0.687‡ |
| Age [years]* | 70.3± 5.3 | 65.7± 7.6 | **0.043**† |
| Symptom duration [years]* | 9.6± 7.9 | 5.2± 4.8 | **0.013**† |
| Standard alcoholic drinks per month [n]^#^ | Males 15 (5-30) Females 2 (0-4) | Males 12 (6-22) Females 0.5 (0-6) | Males 0.850**^⁒^**  Females 0.857**^⁒^** |
| BMI* | 26.6± 4.1 | 28.5± 4.3 | 0.154† |
| Diabetic/Non-diabetic | 2/11 | 8/45 | 0.979‡ |
| **Clinical parameters** | | | |
| MDS-UPDRS III^#^ | 6 (4-15) | 4 (2-8) | **0.026^⁒^** |
| MoCA* | 23.2± 3.3 | 24.0± 2.9 | 0.389† |
| UPSIT* | 18.9± 6.8 | 24.5± 7.7 | **0.045**† |
| SCOPA-AUT* | 13.2± 9.3 | 12.5± 7.9 | 0.783† |
| Putaminal SBR* | 2.2± 0.8 | 3.0± 0.6 | **<0.001**† |
| **Biochemical parameters** | | | |
| Serum ferritin [μg/l] ^#^ | Males 113.3 (60.2-187.6) Females 69.1 (68.1-70.1) | Males 197.7 (112.5-317.6) Females 66.8 (46.3-185.8) | Males 0.076**^⁒^** Females 1.000**^⁒^** |
| Serum transferrin [g/l]* | 2.4± 0.2 | 2.5± 0.4 | 0.277† |
| Serum glucose [mmol/l] ^#^ | 5.3 (4.9-6.1) | 5.1 (4.7-5.6) | 0.3357**^⁒^** |
| CDT_adj_ [%]^#^ | 1.2 (0.9-1.4) | 1.3 (1.1-1.6) | 0.189**^⁒^** |

Significant differences are marked with **bold** text.
*values reported as mean± SD; ^#^ values reported as median (interquartile range); † Student’s t-test; **^⁒^** Mann-Whitney test;‡Chi square test.
BMI- Body mass index; MDS-UPDRS– Movement Disorders Society-Unified Parkinson’s Disease Rating Scale; MoCA– Montreal Cognitive Assessment; n- number; PD– Parkinson’s disease; REM– rapid eye movement; SCOPA-AUT– Scales for Outcomes in Parkinson Disease-Autonomic; UPSIT– University of Pennsylvania Smell Identification Test; SBR- specific binding ratio; CDT- carbohydrate deficient transferrin

**Supplementary Table 2:** Demographic, clinical, and biochemical parameters in iRBD and PD patients included in the study and those excluded for lack of CDT values

|  | **iRBD included (n=72)** | **iRBD excluded (n=11)** | **p-value** | **PD included (n=60)** | **PD excluded (n=9)** | **p-value** |
| --- | --- | --- | --- | --- | --- | --- |
| **Demography** | | | | | | |
| Males/females [n] | 64/8 | 9/2 | 0.502‡ | 37/24 | 5/3 | 0.920‡ |
| Age [years]* | 67.1± 7.3 | 65.8± 4.9 | 0.579 | 65.1± 9.4 | 68.0± 10.0 | 0.414 |
| Symptom duration [years]* | 6.1± 5.6 | 7.4± 6.2 | 0.750† | 1.7± 0.2 | 1.8± 0.3 | 0.946† |
| Standard alcoholic drinks per month [n]^#^ | Males 10.5 (5-22) Females 0.5 (0-4.5) | Males 5.0 (2-30) Females 1 (0.5-1.5) | Males 0.460**^⁒^**  Females 0.781**^⁒^** | Males 20 (10-29) Females 3.5 (0-7.0) | Males 8 (7-30) Females 4.0 (3-17.5) | Male 0.801**^⁒^**  Female 0.389**^⁒^** |
| BMI* | 28.3± 4.3 | 28.4± 4.9 | 0.830† | 28.0± 4.1 | 25.3± 2.7 | 0.292† |
| Diabetic/Non-diabetic | 12/60 | 1/10 | 0.520‡ | 4/57 | 1/7 | 0.542‡ |
| Conversion rate [%] | 18.1 | 9.1 | 0.460‡ | n.a. | n.a. | n.a. |
| **Clinical parameters** | | | | | | |
| MDS-UPDRS III^#^ | 5.5 (2-8.3) | 3.0 (1.5-5.0) | 0.141^⁒^ | 29.0 (21-40) | 22.0 (16-29.5) | 0.058^⁒^ |
| MoCA* | 23.8± 2.9 | 22.2± 3.1 | **0.004†** | 24.7± 3.1 | 24.3± 2.5 | 0.069† |
| UPSIT* | 23.0± 7.9 | 20.5± 9.5 | 0.06† | 21.0± 6.2 | 19.9± 6.2 | **<0.001†** |
| SCOPA-AUT* | 12.5± 8.0 | 8.9± 7.9 | 0.128† | 9.8± 5.2 | 9.4± 5.8 | 0.064† |
| Putaminal SBR* | 2.8± 0.7 | 3.5± 0.8 | **0.013†** | 1.5± 0.4 | 1.4± 0.5 | 0.604† |
| **Biochemical parameters** | | | | | | |
| Serum ferritin [μg/l] ^#^ | Males 169.8 (101.5-275.6) Females 69.1 (57.5-100.7) | Males 137.5 (83.8-316.7) Females 237.0 (173.9-300.2) | Males 0.867**^⁒^** Females 0.117**^⁒^** | Males 166.9 (93.7- 221.0) Females 105.2 (63.1- 136.5) | Males 148.1 (91.1-204.0) Females 59.3 (33.5-77.7) | Males 0.620**^⁒^** Females 0.123**^⁒^** |
| Serum transferrin [g/l]* | 2.5± 0.3 | 2.6± 0.4 | 0.497† | 2.5± 0.3 | 2.6± 0.3 | 0.457† |
| Serum glucose [mmol/l] ^#^ | 5.2 (4.8-5.9) | 5.0 (4.8-5.2) | 0.420**^⁒^** | 5.3 (5- 5.625) | 5.2 (4.8- 5.8) | 0.622**^⁒^** |

Significant differences are marked with **bold** text.
*values reported as mean± SD; ^#^ values reported as median (interquartile range); †ANCOVA with p-values adjusted for age and sex wits post-hoc least square difference test (ANOVA was used for age comparison); **^⁒^** Kruskal-Wallis test with post-hoc Dunn’s test;‡Chi square test.
BMI- Body mass index; MDS-UPDRS– Movement Disorders Society-Unified Parkinson’s Disease Rating Scale; MoCA– Montreal Cognitive Assessment; n- number; PD– Parkinson’s disease; SCOPA-AUT– Scales for Outcomes in Parkinson Disease-Autonomic; UPSIT– University of Pennsylvania Smell Identification Test; n.d. – not done

**Supplementary Fig.3** Between-group comparison and survival analysis in iRBD using lower cutoff (<90% prediction interval) for abnormal DAT-SPECT


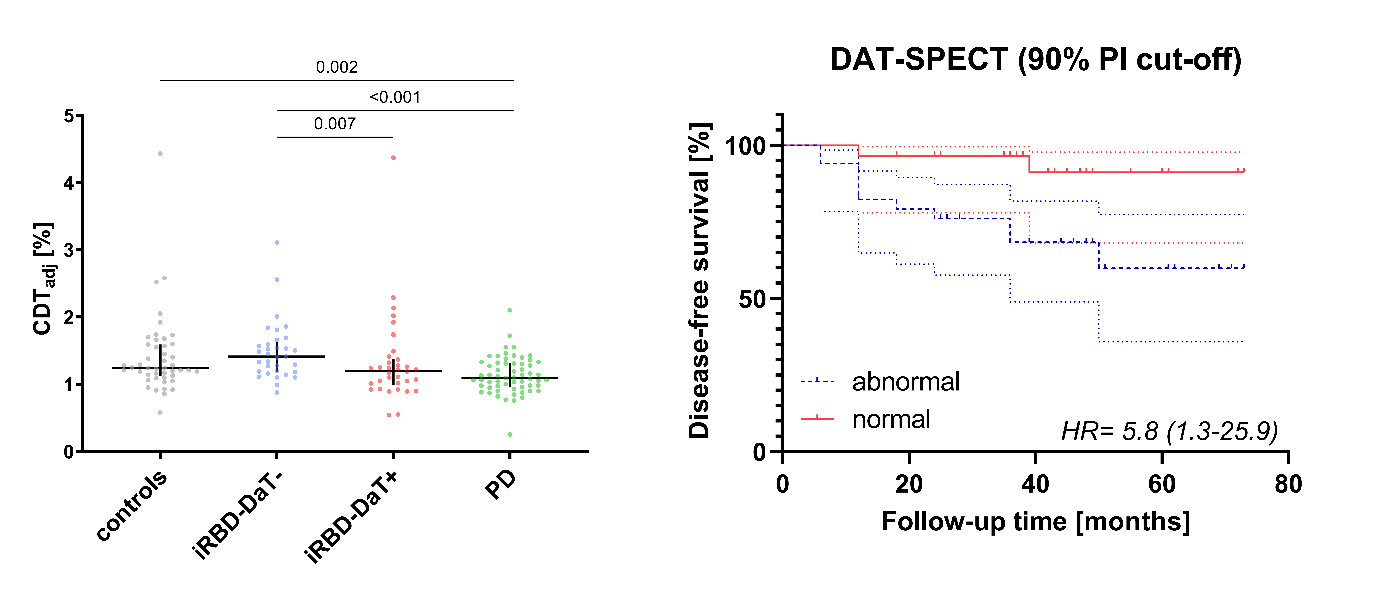
For this analysis, iRBD groups with abnormal DaT-SPECT (iRBD-DaT+)/ normal DaT-SPECT (iRBD-DaT-) were defined as having the lower putaminal SBR from both hemispheres below/ above or equal the 90% prediction interval.
Left image: between-group comparison of CDT_adj_ values was done using Kruskal–Wallis test with pot-hoc Dunn’s test; p-values of post-hoc tests are shown. Horizontal/ vertical lines represent medians/ interquartile ranges.
Right-image: Kaplan-Meier plots for participants with normal and abnormal (i.e., <90% prediction interval) DaT-SPECT. Dotted lines represent 95% confidence bands. Hazard ratio (HR) calculated using Cox proportional hazards regression analysis adjusted for age and sex with 95% confidence interval in parentheses is shown.

**Supplementary Fig.4** Between-group comparison and survival analysis in iRBD using CDT (%) unadjusted for alcohol consumption


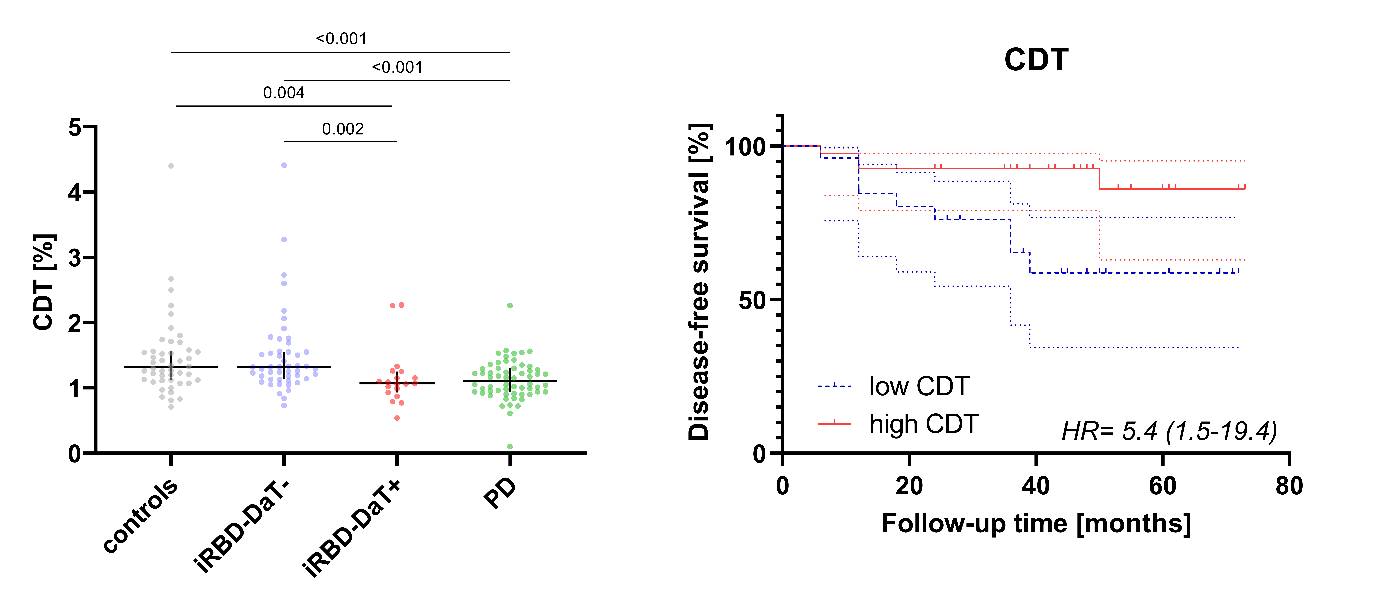
Left image: between-group comparison of raw (unadjusted for alcohol intake) CDT values was done using Kruskal–Wallis test with pot-hoc Dunn’s test; p-values of post-hoc tests are shown. Horizontal/ vertical lines represent medians/ interquartile ranges.
Right image: Kaplan-Meier plots for low (<1.165) and high (≥1.165) unadjusted CDT values. Dotted lines represent 95% confidence bands. Hazard ratio (HR) calculated using Cox proportional hazards regression analysis adjusted for age and sex with 95% confidence interval in parentheses is shown.
